# Case of a Young Lady Who Swallowed a Pin

**Published:** 1785

**Authors:** 


IV. *Caſe of a young lady who ſwallowed a pin. By Mr.* Samuel Gillam Mills, *Surgeon at Greenwich, in Kent.*

NOVEMBER 23d, 1780, I was deſired to viſit Miſs D. a young lady of Greenwich, between nineteen and twenty years of age, of a delicate fibre, and endued with great ſenſibility. She complained of tightneſs of the cheſt, attended with a cough and conſiderable hoarſeneſs. Theſe ſymptoms were ſomewhat relieved by bleeding.

On the 8th of December, ſhe complained of pain in her left ſide, and of ſome hyſterical affections ſhe had been ſubject to. I was informed ſhe never had had a copious diſcharge of the menſtrual flux, and had now miſſed it for ſix weeks.

Imagining that her preſent complaints originated from ſuppreſſed menſes, I was about putting her on a plan that night to excite their difcharge, when in the afternoon they appeared in the accuftomed partial manner.

On the 10th, they ceafed; and in the afternoon fhe was feized with fpafms in her head and face, together with violent hyfteric fits. In the intervals of fenfe, fhe complained of excruciating pain under her left breaft. Once by retching, fhe brought up a tea-fpoonful of coagulated blood mixed with mucus refembling pus, and during the enfuing week fhe was never more than three or four hours at a time free from the fpafms in her head or face, or from the fits. About the 10th day from her feizure, an hydrophobia appeared, and remained near forty-eight hours. During this ftate, fhe expreffed the greateft antipathy to fluids, and when I moiftened her lips with bread dipped in wine or water, it occafioned an univerfal agitation. At the attack of the hydrophobia fhe loft her fenfes, and became foon after deaf. She would then take neither medicine nor nourifhment but from my hands, bore a cheerful countenance, and entertained herfelf with the moft infantile amufements; but on the leaft attempt to alter the horizontal pofition fhe lay in, would cry out from the pain in her fide. It was more than a fortnight from the difapdisappearance of the hydrophobia before she recovered her senses and hearing; and she then recovered them almost suddenly. From the first of her illness to this period, we had not been able to pursue any regular plan of medicine. She had been purged at different times with the neutral salts, joined with bitters, senna, rhubarb, and calomel, as appeared most advantageous. Between the cathartics, the most powerful antispasmodic medicines had been tried, but no good effects were perceptible either from them or from successive blisters.

Soon after the recovery of her senses, she was attacked with spasms of the diaphragm. During the spasms, deglutition was attended with the greatest difficulty; for as often as her resolution impowered her to swallow, the fluid was regurgitated from the stomach. Her breath in these fits was drawn so quick and short, that the sound resulting from such quick inspiration might be distinctly heard in the adjacent rooms, although the chamber door was shut. Bleeding alone removed these spasms, and the operation was obliged to be repeated as often as they occurred. Now and then the loss of little more than a large spoonful of blood would instantly remove the spasm; in general, general, however, the loſs of four ounces was neceſſary, rarely ſix. Theſe ſpaſms returned for many days at a certain hour, and the patient was ſenſible of their approach a conſiderable time before their actual attack, by feeling the pain in her ſide increaſe with a ſenſe of fluttering round the part. At length ſhe became incapable of moving herſelf in bed without being in imminent danger of bringing on the difficulty of breathing; and ſhe frequently complained that her ſide felt as if it were nailed to the bed.

A fortnight had elapſed ſince the firſt attack of the ſpaſms, when her menſes appeared rather more in quantity than uſual: during their flow her breathing was not affected, but immediately it ceaſed the ſpaſms returned. After this, ſhe continued near a month with almoſt daily attacks, ſometimes even twice in the day, when ſhe was ſeized with a ſickneſs at the ſtomach without any apparent cauſe. This ſickneſs would laſt many hours, and it was obſerved, that on the days on which the ſickneſs attacked her ſhe was free from the ſpaſms of the diaphragm. This ſpecies of ſickneſs did not produce thoſe ſpaſms, although any medicine or nouriſhment that induced a nauſea, or retching, almoſt infallibly brought them on: near three weeks paſſed without without the neceffity of bleeding more than twice or thrice ; but after this period the fpafms returned almoft daily for near a fortnight. On the 23d of March, 1781, fhe was attacked with an oppreffion on the cheft, and a confiderable hoarfenefs, fimilar to the complaints I at firft had bled her for. Towards the latter end of January fhe had felt fome fymptoms, which were then afcribed to her having taken cold, and as they then appeared to have brought on the difficulty of breathing, I was now fearful of the fame effect, and was induced to bleed her by way of prevention. In the evening violent retchings came on ; in one of thefe, fhe fcreamed out through pain, and faid fhe had brought up fomething that had hurt her violently. I obferved in the bafin, into which fhe had retched, a fmall quantity of phlegm, interfperfed with pieces of mucus of more than common vifcidity. As the patient perfifted in thinking fhe had brought up fomething in the laft retching that had given her exquifite pain, I broke feveral of thefe pieces of mucus with my forceps, and in one piece found enveloped a crooked middle white pin in a fmall degree tarnifhed. After this fhe paffed a tolerable night, but but the four ſucceeding days were attended with almoſt continual retching.

The pin thus thrown up was known to have been ſwallowed on the 13th of Auguſt, 1779, I was now ſanguine in my expectation that the patient would ſoon recover, particularly as I had for ſome little time before ſuſpected that a pin might produce the unaccountable diſtreſs this young lady ſuffered. I was led to this ſuſpicion from being informed that a pin had been ſwallowed, and from peruſing the caſe, in the Philoſophical Tranſactions (*vol.* 59), of a girl who had ſwallowed three pins, in which caſe I thought I could trace ſymptoms ſimilar to thoſe of my patient.

The ſpaſms of the diaphragm did not return till April 7th, when, upon the patient's receiving a ſudden ſhock in bed, the pain in the ſide increaſed, and the ſhortneſs of breath inſtantly came on.

From April 7th to May 9th, ſhe had only three attacks. On May 8th, ſhe brought up a tea ſpoonful of coagulated blood mixed with matter like mucus. On the 10th, ſhe was ſeized with an oppreſſion of the breath and a hoarſeneſs. The next day in the afternoon, theſe ſymptoms having much increaſed I bled, her.

 Two

Two hours after the bleeding, the ſpaſm of the diaphragm came on, and the operation was obliged to be repeated.

May 14th, the oppreſſion on the breath was much the ſame as on the preceding day. At night the ſpaſm of the diaphragm coming on, ſhe was bled; and immediately after the operation, the oppreſſion on the cheſt, hoarſeneſs, and ſpaſms went entirely off; but the latter returning the next day, ſhe was again bled.

On the 18th, ſhe was removed, for the firſt time ſince her illneſs, into an arm chair. From this day to the 24th of June, ſhe had nine attacks of the ſpaſms of the diaphragm.

In the evening of the 23d of June, ſhe was ſuddenly ſeized with an excruciating pain in her ſide, and likewiſe in the courſe of the ſternum; in half an hour ſhe entirely loſt her ſenſes, and remained in that ſtate three quarters of an hour, when her recovery was as ſudden as the ſeizure.

On the 24th of June, at ſix in the morning, ſhe was removed to the diſtance nearly of a mile, in a machine contrived for the purpoſe. She was brought down ſtairs in a ſtate of inſenſibility from which ſhe rouſed on coming into the air; and the ſhortneſs of breath immediately diately came on, but was quickly removed on her being put to bed and bled. The ten enſuing days were attended with almoſt conſtant retching.

In the removal ſhe caught cold. Her throat inflamed and ſlightly ſuppurated. A new complaint now ſeverely afflicted her, viz. a pain in the left ſide of her head and face; it uſually came on about three in the afternoon, and continued with great violence till ſix or ſeven the next morning. Theſe pains had ſlightly begun to affect her before ſhe was removed; but from the latter end of June till the latter end of Auguſt they were inconceivably ſevere, every day or every other day. It was obvious, that if any topical application relieved the pain in the head and face, the pain in the ſide became immediately augmented, and the ſhortneſs of breath was clearly twice brought on by eaſe procured to the head and face. From July 6th to Auguſt 6th, ſhe was under the neceſſity of being bled ſix times.

In the latter end of Auguſt, the pains in the face began to diminiſh; and in the beginning of September we hoped they had entirely left her. But now ſhe was attacked with violent pain in the left hip, together with ſuch ſpaſms as brought her leg involuntarily into every conceiveable pofture. The warm bath was fo far from affording her any relief, that on the fifth time of her going in fhe was convinced that it increafed inftead of abating the pain.

On the 12th of September, I put her on a courfe of *merc. cal.* and *fulph. aurat. antimon.* On the 1ft of October, this courfe having been duly purfued, the patient was evidently materially relieved, had little or no return of pain, and affumed all the appearances of gradual amendment.

November 12th, fhe complained of pain in the fide on preparing to go to bed, and fell from her chair in a flight fainting fit. The fpafm of the diaphragm inftantly came on for the fecond time fince the 5th of September. The alterative courfe of medicine which had been difcontinued for the laft fortnight, on account of a ficknefs at the ftomach, which came on moft frequently after dinner, was now, at my defire, renewed. Till the 24th fhe continued mending, though under frequent attacks of the ficknefs at the ftomach. On that day, I obferved that her face was fwelled, and the back part of her mouth, in a flight degree, inflamed. I, therefore, fufpended the mercurial courfe, and near three weeks paſt before ſhe recovered from this apparent cold. During that time her face was frequently afflicted with violent pain. She now took, as an alterative, James's powder in ſmall doſes. On the 20th of December, without any known cauſe, the pain in the ſide increaſed, and the ſpaſm of the diaphragm came on.

On Chriſtmas day, ſhe was ſo well recovered as to take an airing (for the firſt time ſince her illneſs) in her carriage, and bore the ride unexpectedly well.

1782. January 14th, ſhe had a ſpaſm of the diaphragm, but till within the two or three preceding days ſhe had rode out every day, and gained ſtrength, though without recovery of appetite or natural ſleep; a ſlight diarrhœa, that had inſenſibly increaſed, made it proper to diſcontinue her powders, which indeed had before often been omitted, either from negligence, or ſome other cauſe. From this day to the 22d, the ſpaſms of the diaphragm came on three times, viz. on the 16th, 17th, and 22d. In the evening of the laſt of thoſe days ſhe complained of a conſiderable tightneſs of the cheſt, which, on the 23d, had increaſed to ſuch a degree, that, at her own requeſt, I bled her, and and had the pleaſure next day to find it had given her conſiderable relief.

January 28th, — for the laſt fortnight ſhe had been much troubled with retchings; theſe had very alarmingly increaſed for the laſt four days, ſo that ſhe could ſcarcely keep down any kind of nouriſhment, even when given in the ſmalleſt quantities. I thought her pulſe weaker than at any time of her illneſs, and much feared ſhe was ſinking. This evening the ſpaſms of the head returned, inſomuch that ſhe was not able, during their continuance, which was many hours, to keep her head one inſtant of time from jerking.

During the courſe of her illneſs ſhe had been accuſtomed to take the thebaic tincture at bed time. The doſe of this medicine had been increaſed to ninety drops; but while ſhe was apparently mending, I had begun to diminiſh it to fifty. I now thought it right, however, to return to the former doſe of ninety drops; and on the 3d of February, as I found the ſpaſms of her head continued at intervals with great violence and with conſtant ſickneſs, I ordered ninety drops of the tincture to be given two hours before the time at which the pain and ſpaſms of the head were expected to return, return, and half that dose to be repeated when they came on.

February 6th, the spasms began to abate; she had taken some nourishment that staid, and was now evidently once again mending; I ordered the opiate to be continued. On the 9th, the spasms of the head were perceived to have gradually abated, but in the afternoon she awoke with considerable pain in the side; and between eleven and twelve at night bleeding, for the last time, became necessary. A gradual amendment now took place; so that on the 10th of April she was enabled to undertake a journey of more than two hundred miles, to the west of England, where, as I had the pleasure to hear, she arrived on the 21st, very little fatigued; the exercise had agreed well with her, had procured her an appetite, and made her sleep at night. She had unremittingly taken the ninety drops of *tinctura thebaica* every night.

Throughout the whole course of this extraordinary illness, the young lady was never free from the pain in the left side, which was invariably fixed between the seventh and eighth ribs, about two inches from the sternum. The shortness of breath was always brought on when the pain in the side had increased to a certain degree, degree, and this increafe of pain, together with the fpafm, has come on in a few minutes from any fudden unexpected noife, fuch as the found of a drum, the report of a gun, flapping of a door, &c. Although fhe lived between two and three hundred yards from the church, the bells, for feveral months, were not allowed to be rung, as they had been proved twice to have induced the fpafm on the breathing. The nervous irritability was at one time fo great, that the dropping of a cinder from the fire has thrown her whole frame into a general tremor. The flighteft motion affected her fide, and it was from December 9, 1780, to May 18th, 1781, before fhe was able to be taken from the bed, and placed on a fofa in any other way than by drawing her in the horizontal pofture fhe lay in, by the affiftance of the under fheet; and even this, though done with all poffible fteadinefs and care, has brought on the fhortnefs of breath: purging motions as well as coftive ones have been followed by thefe fpafms.

To relieve this complaint, various remedies were unfuccefsfully adminiftered; but the frequent and long-continued ficknefs at the ftomach was found an invincible impediment to the daily continuance of medicine. Befides the remedies already already particularly mentioned, at different times, in the courfe of the difeafe, the bark, in fmall and large dofes, flowers of zinc, æther, mufk, and other antifpafmodics. Emetics were alfo repeatedly adminiftered without any material benefit. The common faline draught was found moft ufeful in relieving the ficknefs at the ftomach. Leeches, cupping, and finapifms were tried, but did not prove more advantageous than blifters. Electricity had a long and fair trial; at times fhe thought it relieved her, but, for my own part, I thought it ferved only to amufe her. One fingularity attending its ufe was, that if adminiftered in very flight fhocks, or by drawing fparks or only a very ftrong ftream, it would foon bring on a flux of tears, with a dimnefs threatning deprivation of fight. She has been placed in the warm bath both before and during the attack of the fpafm. When fhe was in the water fhe was relieved, but the fpafm would return on her being removed into bed; fo that it procured only a temporary relief, which was obliged, after a few trials, to be given up, as the moving from the fide of the bed to the tub was attended with fuch excruciating pain, as infallibly brought on the fhortnefs of breath.

 The

The thebaic tincture was given in as large doses as her head and stomach would bear. She has taken, at different periods of her illness, an hundred and fifty drops; but even that, though as much as could be ventured on at a dose, would neither prevent nor sensibly lessen the spasmodic attack. Any medicine that created sickness or nausea would almost certainly bring on the spasms of the diaphragm. Opiates, when taken at bed time, though they did not procure sleep, always induced more or less tranquillity; and I am apt to attribute her recovery to her constantly taking ninety drops of tinctura thebaica at bed time. I am led to this opinion, from having observed that the spasms twice returned when the dose of the opiate, under the idea of her amendment, had been gradually diminished. Although bleeding never failed almost instantly to remove the spasm, it would not prevent the attack; and what appeared to me extraordinary was, that notwithstanding her extreme weakness, it was only once succeeded by fainting. I have sometimes waited five or six hours in hopes of seeing her breathing relieved, without the assistance of the lancet, and then have been obliged to have recourse to it. A sickness at the ftomach, fo that no medicine or nourifh-ment could be given, frequently fucceeded bleeding, and continued for many hours; and often, after the fpafm of the diaphragm had gone off, a locked jaw would come on. From this ftate fhe could fometimes be roufed, by gently infinuating a tea-fpoonful of any grateful liquor between her clinched teeth; at other times fhe would remain one, two, and even three hours before fhe recovered.

Although thirft, and a quick (but never full and hard) pulfe, were conftant attendants throughout her illnefs, there were no other indications of fever, and thefe fymptoms I af-cribed to inanition. It was remarkable that her pulfe was but very little affected by the fpafms, and not in the leaft altered by bleeding. Her fkin was never in a ftate of perfpiration; nor was it ever dry, or exceeding the healthy temperature of heat; but her hands and feet were frequently fo cold as to require the ap-plication of hot bottles and flannel. Her bowels were inclined to coftivenefs, but were never otherwife indifpofed, unlefs by medicine or errors in diet.

The craffamentum of the blood, after having ftood a due time, was very fmall in proportion to the ferum, and generally was tucked up in a button-like form, with a fmall buffy appearance, on the whole very fimilar to what I have often taken from weak pleuritic patients. The menfes appeared twice only in the courfe of her illnefs, at the times ftated in the preceding account.

