# Errata

**Published:** 1785

**Authors:** 


					ERRATA.
Page 49, line 2 (from th? top), for in the courfe of the difeafe, the
bark, read in the courje oj the ckjcaje Jhe took the bark*

				

